# Penetrating needling from Qiuxu (GB40) to Zhaohai (KI6) versus separate needling for post-stroke lower-limb motor dysfunction: an assessor-blinded randomized controlled trial protocol

**DOI:** 10.3389/fneur.2026.1911011

**Published:** 2026-10-21

**Authors:** Jie Zhang, Lin Wang, Zhen Tian, Meng Wang, Jing Fan, Xiaoxiao Liu, Chao Cui, Yulin Qian, Rui Zhang, Yi Liu, Chunxiao Wan

**Affiliations:** 1 First Teaching Hospital of Tianjin University of Traditional Chinese Medicine, Tianjin, China; 2 National Clinical Research Center for Chinese Medicine, Tianjin, China; 3 Tianjin Medical University General Hospital, Tianjin, China; 4 Department of Physical and Rehabilitation Medicine, Tianjin Medical University General Hospital, Tianjin, China

**Keywords:** acupuncture, functional near-infrared spectroscopy, lower-limb motor dysfunction, neurorehabilitation, penetrating needling, randomized controlled trial, stroke, surface electromyography

## Abstract

Post-stroke lower-limb motor dysfunction limits mobility, balance, and independence and remains a major target of neurorehabilitation. Acupuncture is widely used as an adjunctive intervention after stroke, but direct randomized evidence comparing penetrating needling with conventional separate needling at the same acupoints is limited. This protocol describes an active-comparator randomized controlled trial comparing penetrating needling from Qiuxu (GB40) to Zhaohai (KI6) with separate needling for post-stroke lower-limb motor dysfunction, with functional near-infrared spectroscopy (fNIRS) and surface electromyography (sEMG) included as exploratory mechanistic outcomes. This single-center, prospective, assessor-blinded, parallel-group superiority trial will enroll 130 participants with subacute post-stroke lower-limb motor dysfunction. Participants will be randomized 1:1 to penetrating needling from Qiuxu (GB40) to Zhaohai (KI6) or to separate needling at the same acupoints. Both groups will receive 12 acupuncture sessions over 2 weeks, delivered 6 times per week, with 30 min of needle retention per session, together with the same total rehabilitation dose of 30 min/day, 5 days/week. The primary outcome is the Fugl-Meyer Assessment for Lower Extremity (FMA-LE) score at the end of treatment (T1). The primary analysis will use analysis of covariance adjusted for baseline FMA-LE. Secondary clinical outcomes include the Berg Balance Scale, Manual Muscle Testing, and Modified Ashworth Scale. Registered secondary neurophysiological assessments include fNIRS and sEMG; their derived measures will be analyzed exploratorily.

**Clinical trial registration:**

https://itmctr.ccebtcm.org.cn/mgt/project/view/2037483478790045696, ITMCTR2026000773.

## Introduction

Stroke remains a major global cause of disability, and long-term management of post-stroke dysfunction continues to pose a substantial public health challenge (1). Motor impairment is among the most common and clinically important sequelae of stroke, and the extent of motor recovery directly influences activity performance, independence in activities of daily living, and social participation (2, 3). Recovery of lower-limb function is closely linked to mobility, balance, and independence and is therefore a central target of neurorehabilitation (4, 5). Post-stroke lower-limb motor dysfunction is not limited to weakness; it also involves multilevel disturbances in neuromuscular control, including impaired selective motor control, reduced coordination, abnormal muscle synergies, and excessive periarticular co-contraction (6–8). These disturbances jointly constrain walking and functional recovery after stroke and underscore the relevance of sensory input and sensorimotor regulation in post-stroke motor rehabilitation (6–9).

Acupuncture is widely used as an adjunctive intervention in stroke rehabilitation (10). Although acupuncture combined with conventional rehabilitation may improve motor function, spasticity, and activities of daily living in selected post-stroke populations, the current evidence base is limited by variable methodological quality, heterogeneous interventions, and inconsistent reporting (11, 12). Some guidelines and guideline overviews acknowledge acupuncture as a potential adjunctive option for selected post-stroke impairments (13, 14). However, evidence comparing specific needling techniques remains limited, and clinically relevant technique questions should be evaluated using randomized designs rather than assumed from general acupuncture evidence (10, 15).

Penetrating needling from Qiuxu (GB40) to Zhaohai (KI6) and separate needling at Qiuxu and Zhaohai use the same acupoint pair but differ in needle number and specifications, insertion depth, tissue trajectory, and the extent of local stimulation. This trial therefore compares two standardized acupuncture protocols as complete intervention packages; any between-group effect cannot be attributed to needle trajectory alone. Needling depth, tissue layer, and manipulation may modulate local mechanical transduction, peripheral afferent input, and the sensory characteristics of stimulation, providing a plausible basis for comparing these distinct interventions (16–19).

The peri-ankle region, including the sinus tarsi and adjacent tissues, contains abundant sensory nerve endings and mechanoreceptors, including free nerve endings, Ruffini endings, Golgi-like endings, and Pacinian corpuscles (20, 21). This anatomy supports the hypothesis that local needling may influence distal sensory input and sensorimotor regulation (9, 22). For a post-stroke neurorehabilitation protocol, however, clinical improvement alone is not sufficient to understand potential mechanisms. Functional near-infrared spectroscopy (fNIRS) is non-invasive, portable, and tolerant of motor tasks, making it well suited to stroke rehabilitation research (23, 24). Surface electromyography (sEMG) provides objective information about muscle recruitment, co-contraction, and peripheral neuromuscular control (25). Together, fNIRS and sEMG can provide complementary exploratory information on central cortical hemodynamics and peripheral neuromuscular control relevant to lower-limb recovery (23–27).

This protocol goes beyond a registry entry by specifying the clinical intervention, active comparator, acquisition procedures for exploratory fNIRS and sEMG outcomes, and the planned statistical framework. The trial aims to compare, as an adjunct to conventional rehabilitation, the effectiveness of penetrating needling from Qiuxu (GB40) to Zhaohai (KI6) with separate needling at the same acupoints for post-stroke lower-limb motor dysfunction, and to explore whether clinical changes are accompanied by fNIRS- and sEMG-derived changes in central and peripheral measures.

## Methods and analysis

### Study design

This is a single-center, prospective, randomized, assessor-blinded, parallel-group superiority trial. Eligible participants will be allocated in a 1:1 ratio to the experimental or control group. The intervention period will last 2 weeks, with assessments performed at baseline (T0) and after treatment completion (T1). Recruitment commenced on 31 March 2026 and is expected to be completed by 31 December 2026. No trial results are reported in this protocol.

### Patient and public involvement

Patients and the public were not involved in the design of this study. No formal patient or public involvement was planned for the conduct, analysis, or reporting of the trial.

### Setting

The trial will be conducted in the Department of Rehabilitation of First Teaching Hospital of Tianjin University of Traditional Chinese Medicine, Tianjin, China. Outcome assessments, including clinical scales, fNIRS, and sEMG, will be performed in designated assessment rooms within the hospital.

### Eligibility criteria

Inclusion criteria are as follows:

- Age 30–80 years;
- Subacute stroke, defined as 2 weeks to 6 months since onset;
- Presence of lower-limb motor dysfunction;
- Cognitive function sufficient to comply with study procedures, defined as a Mini-Mental State Examination score of at least 24;
- Native Chinese-speaking;
- Right-handedness, as determined by the Edinburgh Handedness Inventory;
- Stable vital signs and ability to complete the study procedures; and
- Provision of written informed consent by the participant or their legally authorized representative.

Exclusion criteria are as follows:

- Anticipated severe adherence problems, as judged by the investigators, that would preclude completion of the study procedures;
- Receipt of acupuncture treatment within the previous week;
- History of epileptic seizures, impaired consciousness, or increased intracranial pressure;
- Severe lower-limb spasticity or severe pain affecting assessment or intervention delivery;
- Severe cardiac disease;
- Women who are pregnant, planning to become pregnant, or breastfeeding;
- Severe psychiatric disorders; and
- Any other condition considered by the investigators to render the participant unsuitable for enrollment.

### Randomization and allocation concealment

An independent statistician will generate the random allocation sequence using R software. Fixed-block randomization with a block size of 4 will assign participants to the experimental or control group in a 1:1 ratio. Group assignments will be placed in sequentially numbered, opaque, sealed envelopes. Allocation information will remain concealed until written informed consent has been obtained and the baseline assessment has been completed. A designated researcher will then open the corresponding envelope in enrollment order. Personnel involved in recruitment and allocation implementation will have no access to the sequence before assignment.

### Blinding and emergency Unblinding

Because of the nature of the intervention, acupuncture practitioners cannot be blinded to group allocation. Participants will be informed that they will receive one of two active acupuncture techniques. Outcome assessors, fNIRS/sEMG acquisition staff, data-entry personnel, and statisticians will remain blinded to group allocation. To maintain blinding, acupuncture practitioners will not participate in outcome assessment, instrument-based data acquisition, data management, or statistical analysis. Statistical analyses will be performed on a masked dataset with treatment groups coded as A and B. Unblinding will be permissible only if knowledge of the allocated intervention is considered essential for immediate clinical management of a serious adverse event or other urgent medical condition. Any unblinding decision will be made by the principal investigator, who may request allocation information from the independent researcher responsible for safekeeping of the randomization sequence. Unblinding will be limited to the affected participant whenever possible, and the date, reason, personnel involved, and outcome of unblinding will be documented.

### Interventions

Acupuncture will be administered by licensed acupuncture physicians holding attending physician rank or above, each meeting the approved protocol requirement of at least 3 years of clinical experience and receiving standardized training for this trial. Standard rehabilitation will be delivered by qualified physical therapists or occupational therapists. The intervention schedule is 12 sessions over 2 weeks, 6 times per week, with 30 min retention. All procedures will be performed with the participant in the supine position and with the affected ankle region and distal lower leg fully exposed. Routine skin disinfection will be performed before needling, and all interventions will use disposable sterile filiform needles.

In the experimental group, Qiuxu (GB40) and Zhaohai (KI6) on the affected side will be selected. Qiuxu is located in the depression anterior and inferior to the lateral malleolus, whereas Zhaohai is located in the depression inferior to the tip of the medial malleolus. After routine disinfection, one disposable sterile filiform needle measuring 0.30 × 75 mm will be inserted at Qiuxu on the affected side. The needle will be inserted at an angle of approximately 15–30° to the skin surface, with the tip directed to Zhaohai and advanced slowly through the anteroinferior ankle soft tissue and subcutaneous pathway. Insertion depth will be adjusted according to local anatomy. Advancement will continue until the needle tip reaches the subcutaneous region corresponding to Zhaohai, without penetrating the skin at Zhaohai. After insertion, routine twirling manipulation will be applied. Deqi will be considered achieved when local soreness, numbness, or distension occurs, with or without radiation to the foot. No quantitative deqi-intensity scale is used. Once deqi is obtained, the needle will be retained for 30 min. During retention, needle manipulation will be performed once every 10 min for approximately 30 s each time. Within each participant, manipulation intensity will be kept as consistent as possible across sessions.

In the control group, Qiuxu (GB40) and Zhaohai (KI6) on the affected side will be selected and needled separately after routine disinfection. Two disposable sterile filiform needles measuring 0.25 × 40 mm will be used, one at each acupoint. Both acupoints will be needled in the conventional manner, with an insertion depth of approximately 15–25 mm adjusted according to local tissue conditions. Routine twirling manipulation will be applied after insertion, with the same local soreness, numbness, or distension used to identify deqi. After deqi is obtained, the needles will be retained for 30 min, with manipulation performed once every 10 min for approximately 30 s each time. The number of treatment sessions, needle retention time, and frequency of manipulation in the control group will match those in the penetrating-needling group. The penetrating needling and conventional separate needling procedures are demonstrated in Supplementary Videos 1 and 2, respectively.

Acupuncture may be suspended or discontinued if a participant develops needle fainting, persistent severe pain, overt bleeding, an enlarging hematoma, signs of local infection, clinical deterioration, or requests withdrawal. Before each acupuncture session, research staff will briefly confirm that the participant is clinically fit to undergo the scheduled intervention. The number of sessions completed by each participant will be recorded throughout the study. Completion of all 12 acupuncture sessions will be regarded as good adherence and documented for subsequent analyses.

### Concomitant rehabilitation

During the study period, both groups will receive the same total standard rehabilitation dose of 30 min per day, 5 days per week, delivered by qualified therapists. The program will emphasize lower-limb motor control, balance, transfers, and gait training according to each participant’s clinical status. Routine medication and other clinically indicated treatment may continue as needed; treatment types and changes during the trial will be recorded. Other physiotherapeutic measures and any additional acupuncture treatment will be documented in detail. Newly initiated invasive interventions targeting the peri-ankle region should be avoided where possible; if such interventions occur, they will be recorded.

### Outcomes

Outcome assessments will be conducted at baseline (T0, before the first treatment session) and after the intervention (T1, within 6 h of completion of the 12th treatment session) (Table 1). The primary outcome is the total Fugl-Meyer Assessment for Lower Extremity (FMA-LE) score at T1, a commonly used measure of post-stroke motor recovery with established measurement properties (28). The primary treatment effect will be estimated as the adjusted between-group mean difference at T1, with baseline FMA-LE score included as a covariate.

**Table 1:** Participant timeline.

Item	Screening/enrollment	Baseline (T0)	Week 1	Week 2	End (T1)
Informed consent	X				
Eligibility screening and demographics	X				
Randomization		X			
Acupuncture intervention (experimental/control)			X (6 sessions)	X (6 sessions)	
PT/OT			X	X	
FMA-LE		X			X
BBS/MMT/MAS		X			X
fNIRS		X			X
sEMG		X			X
AEs/SAEs			X	X	X

PT, physical therapy; OT, occupational therapy; FMA-LE, Fugl-Meyer Assessment for Lower Extremity; BBS, Berg Balance Scale; MMT, Manual Muscle Testing; MAS, Modified Ashworth Scale; fNIRS, functional near-infrared spectroscopy; sEMG, surface electromyography; AEs, adverse events; SAEs, serious adverse events. AEs/SAEs will be recorded throughout the intervention period and summarized at T1.

Secondary clinical outcomes include the Berg Balance Scale (BBS), Manual Muscle Testing (MMT), and Modified Ashworth Scale (MAS), assessed at T0 and T1. The BBS is commonly used in stroke rehabilitation and has supportive measurement evidence (29). Secondary clinical outcomes will be analyzed at T1 with adjustment for the corresponding baseline value, using outcome-appropriate models as specified below.

Registered secondary neurophysiological outcomes include functional near-infrared spectroscopy (fNIRS) and surface electromyography (sEMG); these outcomes will be analyzed exploratorily. fNIRS and sEMG are acquired at T0 and T1 in separate assessment sessions rather than synchronously. For fNIRS, analyses focus on task-related oxygenated hemoglobin (HbO) responses in eight lesion-side-standardized regions of interest (ROIs) and resting-state inter-ROI connectivity. For sEMG, derived measures from bilateral tibialis anterior (TA) and medial gastrocnemius (GAS) during active affected-side ankle dorsiflexion comprise average rectified value (ARV), root mean square (RMS), integrated electromyography (iEMG), and affected/unaffected-side ratios. The sEMG analysis focuses on the TA and GAS ARV affected/unaffected-side ratios; absolute affected-side ARV and iEMG are supportive measures.

### fNIRS acquisition and analysis plan

fNIRS data will be acquired with the NirScan-6000Br system (Danyang Huichuang Medical Equipment Co., Ltd., China): 24 sources, 24 detectors, 63 channels, 11 Hz sampling, and wavelengths of 730, 808, and 850 nm. Recordings at T0 and T1 will comprise an 8-min seated, eyes-closed resting-state assessment and a seated active affected-side ankle-dorsiflexion task. The task paradigm starts with a 10 s baseline followed by five cycles of 20 s task and 20 s rest, for a total of 210 s. The bilateral ROIs are sensorimotor cortex (SM1), supplementary motor area (SMA), prefrontal cortex (PFC), and somatosensory association cortex (SAC). After lesion-side standardization, they are labeled iSM1/cSM1, iSMA/cSMA, iPFC/cPFC, and iSAC/cSAC (i, ipsilesional; c, contralesional). The complete source–detector arrangement, channel numbering, and predefined ROI–channel assignments are provided in Supplementary Figure S1. fNIRS and sEMG are acquired in separate assessment sessions rather than synchronously. In NirSpark v1.8.9, channels with a coefficient of variation (CV) ≥ 20% in raw intensity will be flagged as candidate bad channels and then reviewed manually on the raw waveform. Confirmed bad channels will be set to NA, without numerical interpolation or neighboring-channel substitution. The software motion correction will be set to Auto (std_thr = 6; amp_thr = 0.5), followed by 0.01–0.10 Hz filtering and conversion using the modified Beer–Lambert law with differential pathlength factors (DPF) of 6/6/6 at the three wavelengths. For task-state HbO, the GLM will model each 20-s task block using a boxcar regressor based on the recorded task markers and convolved with an HRF basis function. Gaussian pre-coloring (FWHM = 4 s) and discrete cosine transform (DCT) detrending (cutoff period = 128 s) will be applied. NirSpark Beta (1) is the task coefficient β_task. For each ROI, β_task will be averaged across valid assigned channels. For resting-state HbO, valid channel-to-channel Pearson correlations will be Fisher r-to-z transformed and averaged over valid cross-ROI channel pairs, yielding 28 unique inter-ROI connections among eight ROIs. Pairs involving confirmed bad channels will be treated as missing, with no imputation. No fixed minimum-valid-channel or whole-record bad-channel threshold is applied.

### sEMG acquisition and analysis plan

sEMG will be acquired at T0 and T1 using the MyoMove-EOW system (Shanghai Nuocheng Electric Co., Ltd., China) at 2048 Hz, with a 10–500 Hz acquisition filter and 50 Hz notch filter. Following SENIAM recommendations, disposable electrodes will be placed over bilateral tibialis anterior (TA) and medial gastrocnemius (GAS). Participants will sit with the knee and ankle at approximately 90° and perform five affected-side active dorsiflexion contractions of approximately 3 s each, with natural relaxation between contractions; bilateral muscle signals will be recorded continuously. Raw data will be exported as EDF files. Offline processing will demean the signal, then apply a bidirectional zero-phase fourth-order Butterworth 20–450 Hz bandpass filter. ARV, RMS, and iEMG will be derived from the valid recording window, with affected/unaffected-side ratios calculated for each muscle. The sEMG analysis will focus on TA and GAS ARV affected/unaffected-side ratios, with absolute affected-side ARV and iEMG as supportive indices. Signal and file integrity will be reviewed. If an EDF file is missing or corrupted, a record is incomplete, acquisition fails, or the participant cannot complete the standardized task or yield a defined measure, the corresponding instrument-based assessment will be recorded as unavailable/missing without numerical imputation.

### Sample size

Sample size was based on the T1 FMA-LE endpoint analyzed using baseline-adjusted analysis of covariance (ANCOVA). Chen et al. (12) reported an absolute pooled standardized mean difference of approximately 0.48 for lower-limb Fugl–Meyer scores when comparing acupuncture with conventional treatment. Because this estimate was not derived from a direct comparison between penetrating needling and separate needling, a standardized effect size of *d* = 0.45 was used as a planning assumption. A baseline–follow-up correlation of *ρ* = 0.50 was assumed based on the methodological discussion of Walters et al. (30). Using the approximate two-group ANCOVA sample size formula described by Borm et al. (31),

*n* = 2(*Z*₁₋*α*/₂ + *Z*₁₋*β*)^2^(1 − *ρ*^2^)/*d*^2^, with a two-sided *α* = 0.05 and 80% power, the required sample size was 58.14 participants per group. Allowing for an anticipated 10% dropout rate increased the required sample size to 64.60 participants per group, which was rounded up to 65 participants per group, giving a total planned sample size of 130 participants.

### Recruitment and retention

Potential participants will be screened daily among inpatients in the Department of Rehabilitation. Potentially eligible patients will be approached by the attending physician or a trained member of the research team and provided with detailed study information. Written informed consent will be obtained before any baseline assessment. After written informed consent has been obtained and baseline assessment completed, eligible participants will be enrolled and randomized. To minimize loss to follow-up, all participants will be recruited from the inpatient ward, and the research team will work closely with ward staff throughout the study. Reasons for missed assessments, treatment discontinuation, protocol deviations, or withdrawal will be documented. Participants who discontinue the allocated intervention or have protocol deviations will still be encouraged to complete outcome assessments wherever possible.

### Data management

All trial data will be collected at prespecified visits using standardized case report forms and source documents. Clinical outcomes will be assessed by trained evaluators using uniform administration procedures and scoring criteria. Scale-based outcomes will be recorded immediately after assessment on paper forms and entered into standardized electronic forms for verification. Source data will be entered independently by two trained data managers into the web-based ResMan Clinical Research Management System. Discrepancies will be checked against source documents and resolved by authorized study personnel. Prespecified coding rules, range checks, logic checks, and missing-data prompts will be embedded in the database to improve data quality. Each participant will be assigned a unique study identification number. The file linking personal identifiers to study identification numbers will be stored separately from the analytical database with restricted access. Paper records will be stored in locked filing cabinets in a secure research office. Electronic data will be stored on secure institutional servers and backed up regularly. Instrument data, including raw and exported fNIRS and sEMG files, will be stored in structured folders identified only by study ID and visit time point. Before database lock, the database will undergo data cleaning, consistency checks, outlier review, and resolution of outstanding queries. After database lock, analyses will be performed on the blinded dataset in accordance with the prespecified statistical analysis plan.

### Statistical analysis

Analyses will be finalized before database lock and unblinding and conducted using R and/or SPSS. All tests will be two-sided, with *p* < 0.05 for the single primary endpoint. Effect estimates will be presented with 95% confidence intervals. Baseline characteristics will be summarized by randomized group as mean ± standard deviation or median (interquartile range) for continuous variables and frequency (percentage) for categorical variables, without baseline significance tests.

The primary analysis will follow the intention-to-treat principle and will include all randomized participants analyzed according to their allocated group. The per-protocol population, defined as participants who complete the allocated intervention without major protocol violations and undergo the primary endpoint assessment within the permitted time window, will be used for sensitivity analyses. The safety analysis set will include all participants who receive at least one treatment session and will be analyzed according to the treatment actually received.

For the primary outcome, ANCOVA will model T1 FMA-LE as the dependent variable, group as the fixed effect, and baseline FMA-LE as a covariate; the treatment effect will be the adjusted between-group mean difference with 95% confidence interval. BBS will use the same baseline-adjusted ANCOVA framework. For ordinal MMT and MAS scores, the analysis will use baseline-adjusted ordinal logistic regression and report group odds ratios with 95% confidence intervals; if sparse categories prevent stable estimation, results will be summarized descriptively and the model limitation reported. Clinical secondary analyses will be supportive.

Secondary fNIRS and sEMG outcomes will be analyzed exploratorily after the acquisition, preprocessing, and quality-control steps described above. The two sEMG ARV affected/unaffected-side ratios, eight task-state fNIRS ROI β_task values, and 28 resting-state inter-ROI Fisher-z connectivity values will form distinct exploratory comparison families. T1 between-group comparisons will use the corresponding baseline value as a covariate when both visits yield valid data. Benjamini–Hochberg false-discovery-rate adjustment will be applied within each exploratory family; estimates and unadjusted 95% confidence intervals will also be shown. Instrument-based findings will be interpreted as supportive and hypothesis-generating.

Missing data will be described by extent, pattern, and reason. For the primary intention-to-treat analysis, missing clinical FMA-LE scores will be addressed by multiple imputation under a missing-at-random assumption, with complete-case and per-protocol analyses reported as sensitivity analyses. Missing instrument-based assessments caused by absent or corrupted files, incomplete recordings, technical failure, inadequate signal, inability to complete the standardized task, or failure to derive the defined index will be recorded as unavailable/missing and will not be numerically imputed. Confirmed fNIRS bad channels and channel pairs will remain missing without interpolation or neighboring-channel substitution. No interim efficacy analysis is planned. No multiplicity adjustment will be applied to the single primary endpoint.

### Adverse events

Adverse events and serious adverse events will be actively checked before, during, and after acupuncture sessions and summarized at T1. Monitored acupuncture-related events include persistent or severe pain, local bleeding, subcutaneous hematoma, needle-related syncope, local infection, persistent sensory abnormality, and suspected nerve injury. For each event, severity, management, outcome, relatedness to treatment, and any treatment interruption, discontinuation, or withdrawal will be recorded. Adverse events and serious adverse events will be summarized by group as frequencies and percentages. Serious adverse events and study-related safety concerns will be reported to the principal investigator and ethics committee according to institutional requirements.

### Trial monitoring

Because this is a single-center, investigator-initiated randomized controlled trial with a relatively small sample size, an acupuncture intervention of overall manageable risk, and no planned interim efficacy analysis, no independent data monitoring committee will be established. Safety monitoring and trial oversight will instead be undertaken by the principal investigator, the research team, and the hospital ethics committee in accordance with prespecified procedures. Trial oversight will cover informed consent, eligibility assessment, randomization and allocation concealment, intervention delivery, outcome assessment, protocol deviations, adverse-event reporting, and verification of consistency between source documents and case report forms. Standardized training based on standard operating procedures will be completed by all study personnel before study initiation. The trial may be suspended or terminated early if unacceptable safety risks, serious ethical concerns, or other circumstances make continuation inappropriate.

## Discussion

This protocol describes a post-stroke neurorehabilitation randomized controlled trial comparing two standardized active acupuncture protocols at the same acupoint pair and under the same rehabilitation background. The trial estimates the comparative effectiveness of penetrating needling from Qiuxu (GB40) to Zhaohai (KI6) versus separate needling, rather than acupuncture versus no acupuncture or sham treatment. The arms differ in needle number and specifications, insertion depth, tissue trajectory, and local stimulation extent. Any observed between-group effect therefore represents the overall difference between the two acupuncture packages and cannot isolate needle trajectory as an independent cause.

Practitioner blinding is impossible because the two needling techniques require different procedures. The protocol therefore uses assessor, fNIRS/sEMG acquisition staff, data-entry, and statistician blinding, masked group coding for analysis, and separation of treatment delivery from outcome assessment to minimize measurement and analytical bias. Participants will be informed that both groups receive active acupuncture techniques.

The inclusion of fNIRS and sEMG is intended to strengthen the mechanistic value of the trial while preserving a clinically interpretable primary endpoint. fNIRS may capture exploratory cortical hemodynamic and connectivity changes in motor-related regions during resting-state and ankle dorsiflexion paradigms, whereas sEMG may capture changes in distal muscle recruitment and co-contraction. Recent multimodal studies have further illustrated the potential value of integrating cortical hemodynamic and muscle activity measures to characterize brain–muscle interactions in stroke survivors and during motor force generation in older adults (32, 33). These registered secondary outcomes will be analyzed exploratorily rather than as confirmatory endpoints because the trial is powered for the clinical FMA-LE endpoint, and the acquisition and preprocessing choices for neurophysiological signals may influence derived measures. Mechanistic findings will therefore be interpreted as hypothesis-generating evidence that may inform future neurorehabilitation studies.

If completed as planned, this trial may inform neurorehabilitation decisions by comparing two reproducible acupuncture protocols under matched treatment frequency, retention time, rehabilitation dose, outcome assessment, and the statistical analysis plan. Central and peripheral exploratory measures may complement, but do not replace, the patient-relevant clinical endpoint.

Several limitations should be considered. The study is single-center, which may limit generalizability to other rehabilitation settings. The intervention period is short, and outcomes are assessed immediately after the 2-week treatment course, so longer-term effects will not be established by this protocol. Practitioner blinding is not possible. Differences between the acupuncture arms involve several coupled procedural components; a group difference cannot be assigned solely to needle trajectory. This two-arm design cannot estimate the specific effect of acupuncture relative to no acupuncture or sham treatment and cannot provide healthy-participant neurophysiological reference values. The fNIRS and sEMG endpoints are exploratory and may be affected by technical noise, task performance variability, and preprocessing decisions. These limitations will guide interpretation of the findings.

## Supplementary material

SUPPLEMENTARY FIGURE S1 fNIRS probe layout, ROI-channel assignments, and acquisition paradigms.

SUPPLEMENTARY VIDEO 1 Video demonstration of the penetrating needling procedure from Qiuxu (GB40) to Zhaohai (KI6).

SUPPLEMENTARY VIDEO 2 Video demonstration of the conventional separate needling procedure at Qiuxu (GB 40) and Zhaohai (KI 6).
